# Morphogenesis and evolution mechanisms of bacterially-induced struvite

**DOI:** 10.1038/s41598-020-80718-y

**Published:** 2021-01-08

**Authors:** Tian-Lei Zhao, Han Li, Hao-Fan Jiang, Qi-Zhi Yao, Ying Huang, Gen-Tao Zhou

**Affiliations:** 1 CAS Key Laboratory of Crust-Mantle Materials and Environments, School of Earth and Space Sciences, University of Science and Technology of China, Hefei, 230026 People’s Republic of China; 2 School of Chemistry and Materials Science, University of Science and Technology of China, Hefei, 230026 People’s Republic of China; 3 State Key Laboratory of Microbial Resources, Institute of Microbiology, Chinese Academy of Sciences, Beijing, 100101 People’s Republic of China; 4 CAS Center for Excellence in Comparative Planetology, University of Science and Technology of China, Hefei, 230026 People’s Republic of China

**Keywords:** Mineralogy, Biomineralization

## Abstract

Bacteria are able to induce struvite precipitation, and modify struvite morphology, leading to the mineral with various growth habits. However, the relevant work involving the morphogenesis is limited, thereby obstructing our understanding of bacterially mediated struvite mineralization. Here, an actinomycete *Microbacterium marinum *sp. nov*.* H207 was chosen to study its effect on struvite morphology. A combination of bacterial mineralization and biomimetic mineralization techniques was adopted. The bacterial mineralization results showed that strain H207 could induce the formation of struvite with grouping structure (i.e., a small coffin-like crystal grown on a large trapezoid-like substrate crystal), and the overgrowth structure gradually disappeared, while the substrate crystal further evolved into coffin-like, and quadrangular tabular morphology with time. The biomimetic experiments with different organic components confirmed that the soluble macromolecules rich in electronegative carboxyl groups secreted by strain H207 dominate the formation of the struvite grouping. The time-course biomimetic experiments with supernatant testified that the increase in pH and NH_4_^+^ content promoted the evolution of crystal habits. Moreover, the evolution process of substrate crystal can be divided into two stages. At the first stage, the crystal grew along the crystallographic *b* axis. At the later stage, coupled dissolution–precipitation process occurred, and the crystals grew along the corners (i.e., [110] and [1-10] directions). In the case of dissolution, it was also found that the (00-1) face of substrate crystal preferentially dissolved, which results from the low initial phosphate content and high PO_4_^3−^ density on this face. As a result, present work can provide a deeper insight into bio-struvite mineralization.

## Introduction

Struvite is a phosphate mineral with a chemical composition of MgNH_4_PO_4_·6H_2_O. It has been discovered in some peculiar natural environments associated with organic matter decomposition, such as guano deposits, basaltic caves, and marshlands^1,2^. Meanwhile, struvite is a prime scale in sewage and wastewater treatment systems as well as a main component of infectious urinary stones in human and animal bodies, and the later aroused the interest of scientists to this mineral^3–6^. Early studies found that the formation of struvite stone was associated with a urinary tract infection caused by urease-producing bacteria such as *Proteus*, *Klebsiella*, *Pseudomonas* and *Staphylococcus* species^3,7,8^, and thus recognizing the link between bacterial activity and struvite formation. Since then, the bacterial mineralization of struvite has received increasing attention.

It has been found that many bacterial strains from different natural habitats are able to induce struvite mineralization^9–19^. Rivadeneyra et al.^9^ isolated 161 aerobic bacterial strains from soil and freshwater, and found that more than half of these strains had the ability to produce struvite crystals. Anaerobic sulfate reducing bacteria (SRB) isolated from river sediment and dolostone could also mineralize struvite^14,15^. At present, it has been recognized that the bacterium-promoted struvite formation is mainly through its degradation of nitrogenous organic compounds, leading to the increase in environmental ammonium content and pH, thereby creating the conditions conducive to struvite formation. Moreover, the biogenic struvite usually have distinctive morphologies, for instance, coffin-, X-like, dendritic, and prismatic shapes^11,12,16^, which are different from the rod-, needle-like, and long tabular shapes formed under neat inorganic conditions^3,20,21^. These indicate that bacteria should exert different effects on the mineralization and morphogenesis of struvite.

However, bacterial growth and metabolism would produce a variety of organic components (e.g., bacterial cell, extracellular polymeric substances (EPS), low molecular weight polypeptide (LMWP), and amino acid), and concomitantly lead to change in solution chemistry (e.g., pH, ammonium and phosphate contents)^16,22–26^. It makes the complexity of the mechanism by which bacteria regulate struvite morphogenesis. As such, a little involving bio-struvite morphogenesis was carried out, although there has been a lot of work reported on bacterial mineralization of struvite. For example, Sadowski et al*.*^27^ found that the struvite obtained from artificial urine with or without *Proteus mirabilis* both exhibited coffin-like structure, but the exposed crystal faces of the two crystals were different, which were explained by electrostatic interactions between negatively charged residues of bacterial cells and crystal surfaces^27,28^. Besides, X-shaped struvite was also observed at high pH value in the presence of *Proteus mirabilis*, and the authors suggested that rapid change in pH value caused by the strain play the main role in the formation of this feature^27^. Our group recently investigated the effects of bacterial cells (*Shewanella oneidensis* MR-1) and its metabolites (i.e., soluble EPS, bound EPS, and low molecular-weight compounds) on the morphogenesis of struvite, and found that the low molecular weight components secreted by the bacterial strain dominated the formation of coffin-like struvite^16^. These results indicate that the mechanism of struvite morphogenesis may be distinguishable in different bacterial mineralization systems.

Herein, *Microbacterium marinum *sp. nov*.* H207 was used to study its effect on struvite morphology. This strain was chosen because (1) it has been demonstrated that the bacterium has a prominent ability to mineralize struvite^19^, (2) it belongs to phylum Actinomycetes, thus can be compared with the strains of phylum Proteobacteria (e.g., *Proteus mirabilis*, *Shewanella oneidensis* MR-1) on regulating morphogenesis of struvite. The bacterial and biomimetic mineralization strategies were used in combination. As a result, the factors that mediate the bio-struvite morphogenesis and evolution were identified, and relevant mechanisms were proposed. This proves useful for a better understanding of struvite biomineralization.

## Results and discussion

### Morphological feature of struvite induced by strain H207

**Figure 1:**
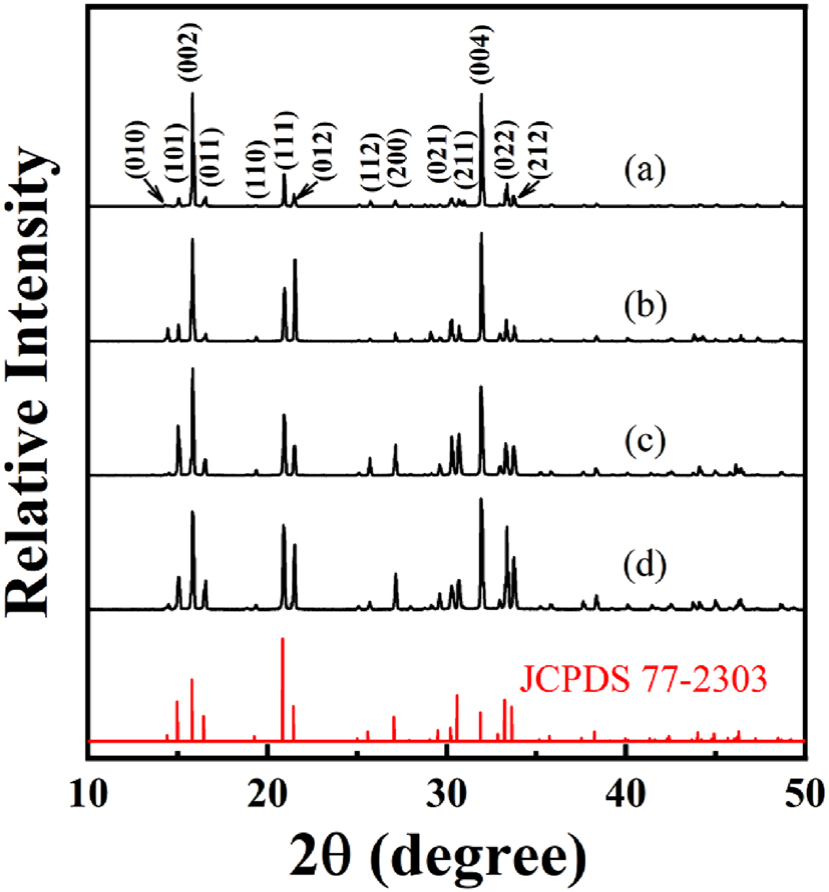
Representative XRD patterns of the samples obtained at a mineralization time of 3 (**a**), 5 (**b**), 8 (**c**), or 15 d (**d**).

**Figure 2:**
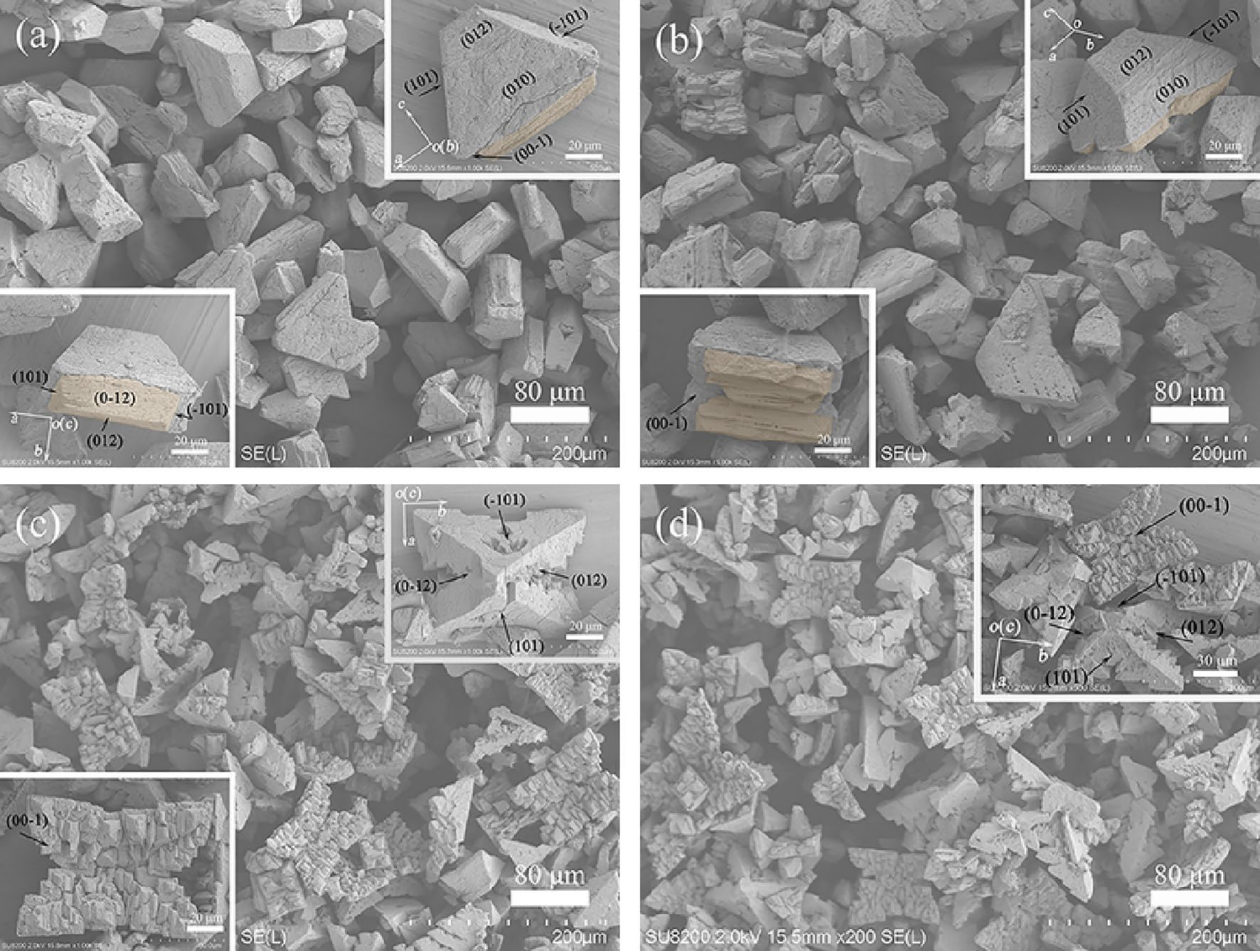
Typical FESEM images of the struvite obtained at a mineralization time of 3 (**a**), 5 (**b**), 8 (**c**), or 15 d (**d**). The overgrown crystals were colored with orange, and the same coloring was also adopted in following figures.

A series of time-course bacterial mineralization experiments were first carried out to understand the morphogenesis and evolution of struvite. At the end of 3 days of incubation, white precipitates appeared in the mineralization experiments, and were collected. Figure 1a shows the representative XRD pattern of the precipitates. The XRD results identified that the products were phase pure orthorhombic struvite with lattice parameters *a* = 6.945 Å, *b* = 6.135 Å, *c* = 11.208 Å, and space group *P*mn2_1_ (JCPDS 77-2303). Moreover, all diffraction peaks were strong and sharp, indicating that the as-obtained struvite was well crystallized. Morphology and texture of the precipitates were further observed by FESEM. The panorama images (e.g., Fig. 2a) showed that the struvite exhibited trapezoid-like morphology, which is consistent with its inherently hemimorphic structure (space group *P*mn2_1_)^11,16,29^. Nevertheless, the magnified SEM images (Fig. 2a insets) displayed that the individual is a combination (i.e., a grouping structure) consisting of large trapezoid-shaped and small coffin-like subunits (colored with orange), and the trapezoid-shaped subunit was composed of the crystal forms pedion {00-1}, domes {101} and {012}, and pinacoid {010} (Fig. 2a upper right inset), while the coffin-like subunit exposed its {101}and {012} domes (Fig. 2a lower left inset), with adopting (00-1) faces as their contact face. Struvite crystals with single crystal and twin crystal habits have been found under the bacterial mineralization system, but to the best our knowledge, such crystal grouping has not been observed, indicating that strain H207 can mineralize struvite with the distinctive morphology and structure. The similar grouping has also been observed in the biomimetic mineralization of struvite with pyrophosphate, namely one frustum-like small crystal grown on the (001) face and two rocket-like small crystals grown on the (00-1) face of each large rocket-like crystal^30^. Moreover, both subunits of the struvite groupings in previous and present work attached along coherent crystallographic direction, i.e., the *c*-axis. Given the significant difference between the size of these two subunits in our case, it can be believed that the small crystals were likely to homoepitaxially grow on the large crystals during the biomineralization.

With the mineralization lasting for 5 days, a marked change in crystal habit was observed (e.g., Fig. 2b). Specifically, the products mainly exhibited coffin-like habit with some fragmented crystals grown on its (00-1) face (Fig. 2b insets). The XRD analysis confirmed that the products were still struvite (e.g., Fig. 1b). The coffin-like habit is a typical struvite morphology, which have been obtained in artificial urine^27,30^, and in the presence of bacteria *Proteus mirabilis* or *Shewanella oneidensis* MR-1^11,16^. However, note that these coffin-like crystal individuals still consisted of the same crystallographic crystal forms as the trapezoid-shaped crystals (Fig. 2a), but the enhanced expressions of the {012}, {101} and {00-1} faces, and weakened expressions of the {010} faces can be recognized (Fig. 2b upper right inset), i.e., the later significantly elongated in the *b* axis relative to the former (Fig. 2a,b). It indicates that the struvite crystal could preferentially grow along the [010] direction with time. The gradual expansion of (00-1) face in trapezoid-like substrate crystal along the [010] direction could cause splitting of the primary small coffin-like crystal, in turn leading to the fragmented crystals (Fig. 2b lower left inset). Further extending the mineralization time to 8 days, a quadrangular tabular struvite was developed (Figs. 1c, 2c). The magnification observations found that the quadrangular tabular crystals possessed {101}, {012}, and {00-1} faces, but the tabular surfaces no longer smooth, that is, emergence of evident pits on (00-1) face and equivalent splitting of {012} faces occurred (insets in Fig. 2c). In contrast to the coffin-like crystals (Fig. 2b), the quadrangular tabular crystals lacked the {010} faces, and the expressions of other crystal faces were all enhanced, indicative of the further growth of struvite crystals along the *b*-axis. Moreover, it is worth noting that no overgrown crystal can be seen on the (00-1) face at this stage (Fig. 2c lower left inset), and since then, no further significant variation in struvite morphology could be recognized, even if the mineralization time was extended to 15 days (Figs. 1d, 2d). Therefore, the bacterial mineralization experiments revealed that the strain H207 could induce the formation of struvite crystal grouping by an overgrowth process, and that the grouping structure could further evolve into relative stable quadrangular tabular structure under current biomineralization conditions.

### Factors responsible for the crystal grouping of struvite

**Figure 3:**
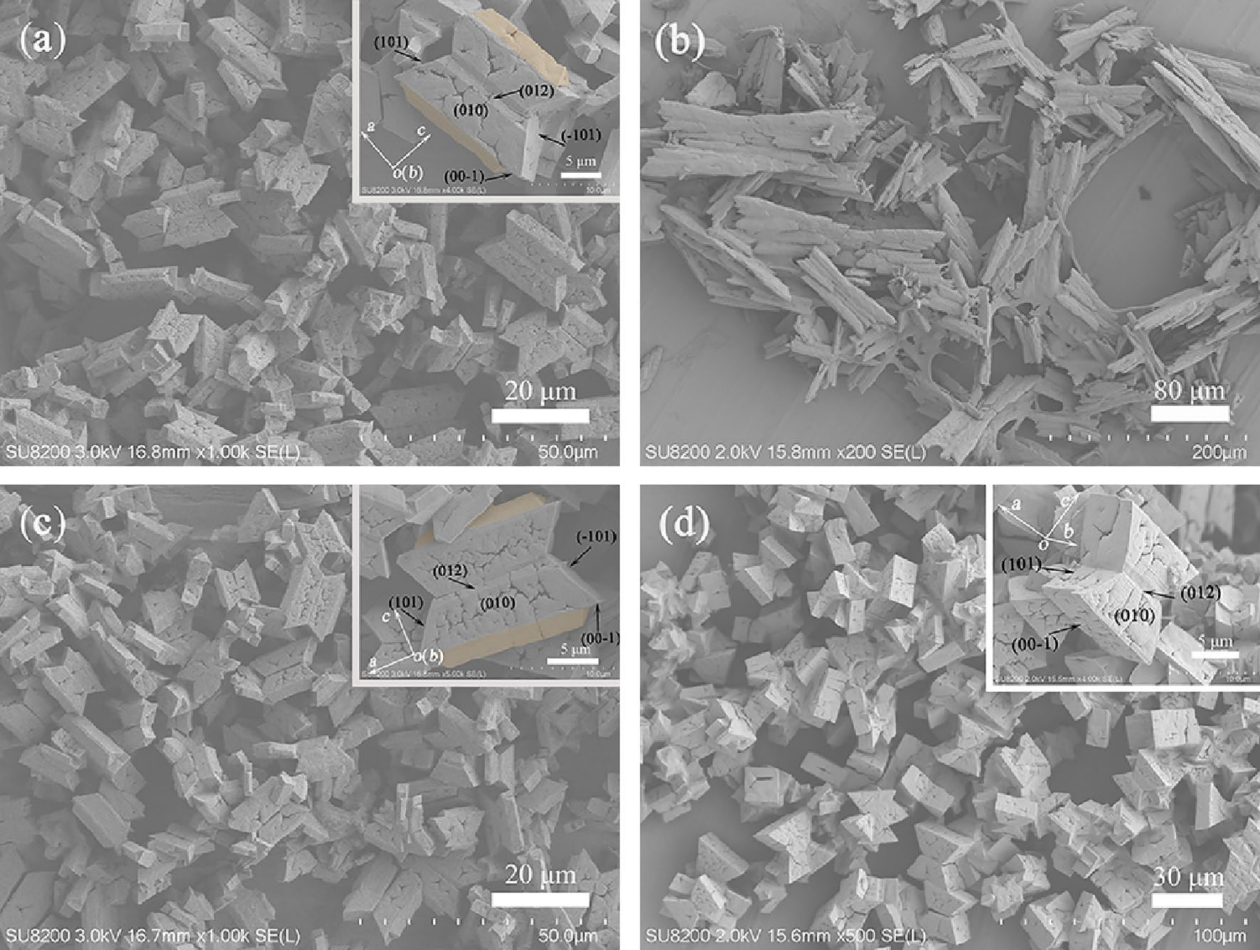
Representative FESEM images of the samples biomimetically synthesized for 30 min with unseparated liquid culture (**a**), bacterial cells (**b**), supernatant (**c**), or uninoculated culture medium (**d**).

In order to unveil the specific factors controlling the struvite morphogenesis, the biomimetic mineralization experiments with different bacterial components were first carried out. Figure 3a depicts the typical FESEM image of the product obtained at a 30 min of mineralization with unseparated liquid culture, exhibiting the presence of twin crystals consisting of trapezoid-like individuals, and the XRD analysis confirmed them as struvite (Supplementary Fig. S1a). The magnification observations found that each trapezoid-like individual exposed {012}, {010}, {101}, and {00-1} faces (Fig. 3a inset), which had the same crystal forms as that obtained by bacterial mineralization for 3 d (Fig. 2a). Meanwhile, a small coffin-like crystal always attach to the (00-1) face of each trapezoid-like individual (Fig. 3a inset, colored with orange). These results indicate that the biomimetic experiment with unseparated liquid culture could obtain the crystal grouping similar to that in the bacterial mineralization experiments. The twin crystal formation in the biomimetic system could be related to the rapid nucleation and growth of struvite (relative to the in-situ bacterial system). This was confirmed by the biomimetic runs with unseparated liquid culture in the presence of lower concentration of ammonia water (1 mmol/L), i.e., only the grouping structure consisting of large trapezoidal and small coffin-like struvite crystals were obtained at 2 h of mineralization (Supplementary Fig. S2). Subsequently, the effect of the supernatant and bacterial cells separated from the unseparated liquid culture on struvite morphogenesis was further investigated under same biomimetic conditions, respectively. At 30 min of mineralization, dendritic struvite crystals were obtained in the solution bearing bacterial cells (Fig. 3b and Supplementary Fig. S1b), excluding the contribution of the bacterial cells to the grouping structures. By contrast, the struvite from the supernatant (Fig. 3c and Supplementary Fig. S1c) exhibited the similar growth morphology to that from the unseparated liquid culture (Fig. 3a). It appears that the components in the supernatant are responsible for the crystal grouping of the bio-struvite. Note that the supernatant contains not only bacterial metabolites, but also possible residue of the initial culture medium. In this context, the control experiment with uninoculated culture medium was also executed to understand the potential effects of culture medium component on bio-struvite morphogenesis. Interestingly, the exclusive struvite twin crystals were derived in this case, and the twins were also composed of trapezoid-like individuals (Fig. 3d and its inset), but lack of the overgrowth structure compared with the supernatant runs (e.g., Fig. 3c). Therefore, it can be concluded that the bacterial metabolites should exert a key control role in the formation of the grouping structure.

**Figure 4:**
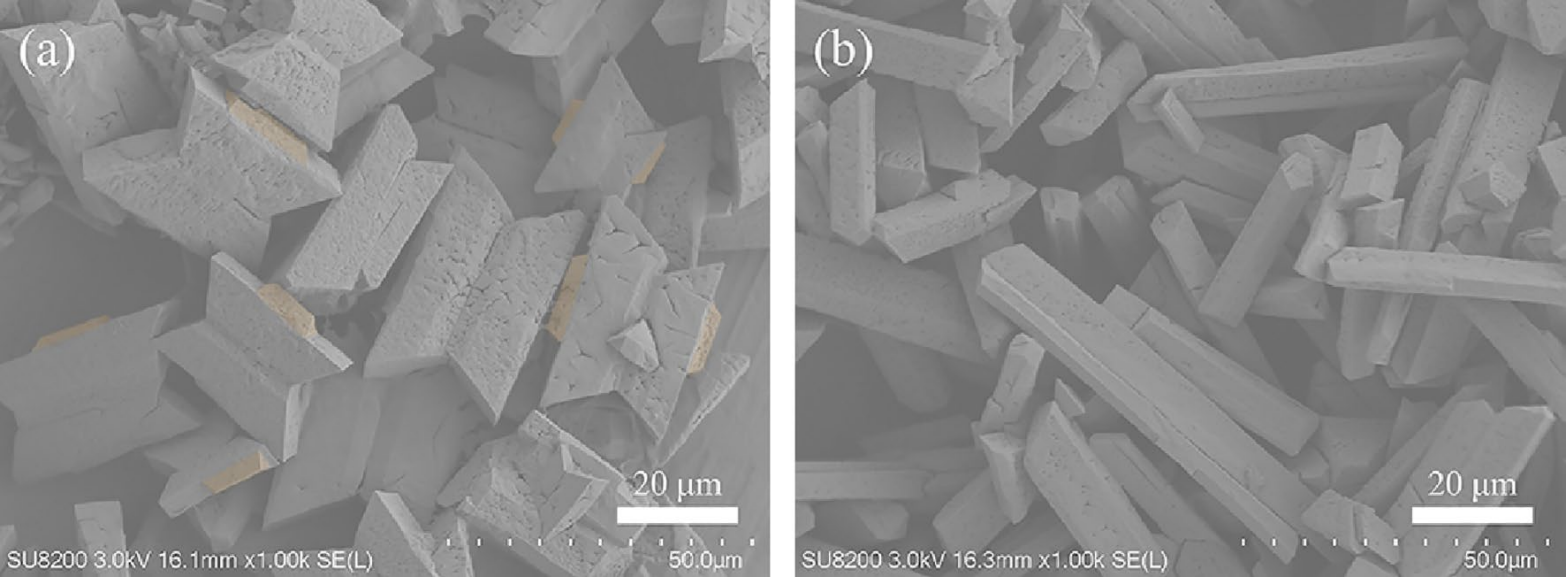
FESEM images of the samples biomimetically synthesized for 30 min with the macromolecule (**a**) or LMW component (**b**) separated from the supernatant.

It is well known that bacterial supernatant contains various organics including polysaccharides, proteins, small molecular organic acid and amino acids, etc^16,22–25^. These biological/organic molecules can potentially influence biomineral shapes^31–34^. To further identify the components that play the role in formation of the grouping, the supernatant was separated into low molecular-weight (LMW) and macromolecule components by ultrafiltration, and the effects of both components on struvite mineralization were subsequently tested. Our results showed that the products obtained in macromolecule component (Fig. 4a) inherited the morphological structure in the supernatant (Fig. 3c), while rod-like struvite (Fig. 4b) was crystallized from mineralization solution with the LMW component. These demonstrated that the soluble macromolecule component secreted by the strain H207 dominates the formation of the grouping structure. Sadowski^27^ found that the coffin-like struvite crystals obtained with and without strain *Proteus mirabilis* have different crystallographic crystal forms, i.e., the coffin-like struvite exposed {012}, {101}, and {00-1} faces in the abiogenic counterpart, while the {012} faces were replaced by the {011} faces in the presence of this strain, and they pointed out that negatively charged residues of bacterial cells may electrostatically interact with the NH_4_^+^ groups on the {011} surfaces, thus leading to an enhanced representation of these faces^28^. Li et al.^16^ confirmed that the negatively charged carboxyl groups in the low molecular-weight component secreted by strain MR-1 play the main role in the formation of biogenic coffin-like struvite, and believed that the preferential binding of the carboxyl groups to (00-1) face of struvite results in an enhanced expression of this face and thus the formation of coffin-like struvite. These results reflect the diversity in regulation of struvite morphology by different strains.

### Factors controlling morphological evolution of struvite

**Figure 5:**
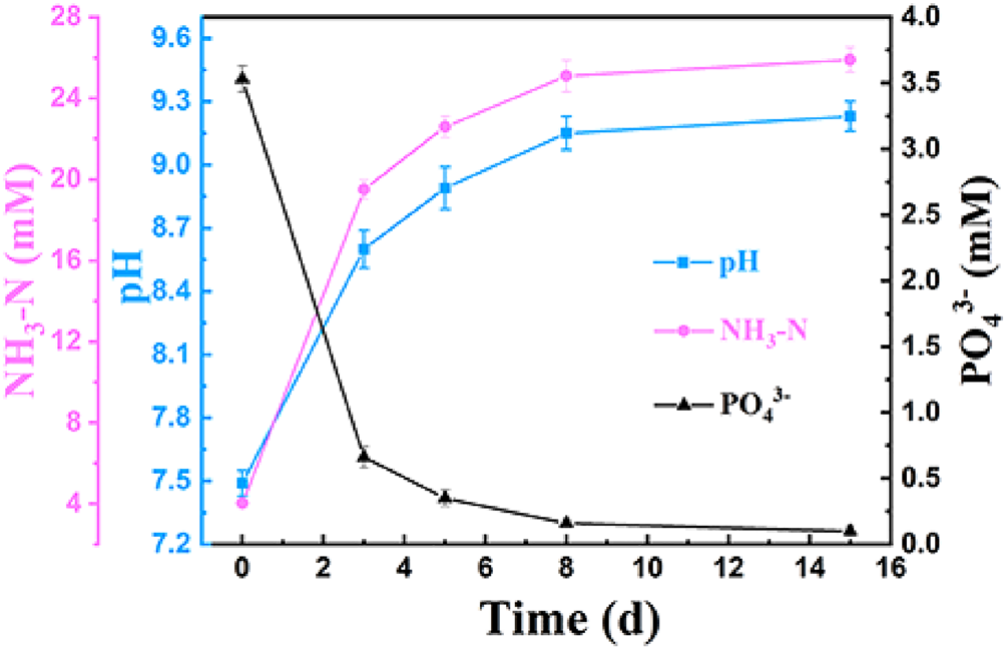
Time evolution of pH, NH_3_-N, and PO_4_^3−^-P contents in bacterial mineralization experiments.

The morphological evolution of struvite from the trapezoid-like, through coffin-like to quadrangular tabular structures (Fig. 2) occurred in time-course bacterial mineralization experiments. Concomitantly, the yield of bio-struvite also observably increased, i.e., from 0.38 (3 d), 0.44 (5 d), and 0.49 g/L (8 d) to 0.51 g/L (15 d) (Supplementary Fig. S2). The increase of struvite yield may be attributed to the rise in the medium pH, ammonium or phosphate content. Therefore, these medium parameters at different mineralization stages were measured. It can be seen from Fig. 5 that the concentration of phosphate decreased over the cultivation, which results from the phosphorus bioutilization and struvite mineralization^12,19^. Conversely, the pH and NH_3_–N content are increasing due to the biodegradation of nitrogenous organics releasing ammonia, and thus elevating pH^17,35^. Moreover, Fig. 5 clearly showed that after 8 day of mineralization, the increases in pH value and NH_3_-N content as well as the decrease in phosphate content became more gentle, and being consistent with the change of struvite yield (Supplementary Fig. S3). This should be attributed to the decrease in metabolic activity of this strain with time. However, it is worth noting that the morphology of struvite remains almost unchanged after that (Fig. 2c,d), indicating that the variation in solution chemistry dominates the morphological evolutionary.

**Figure 6:**
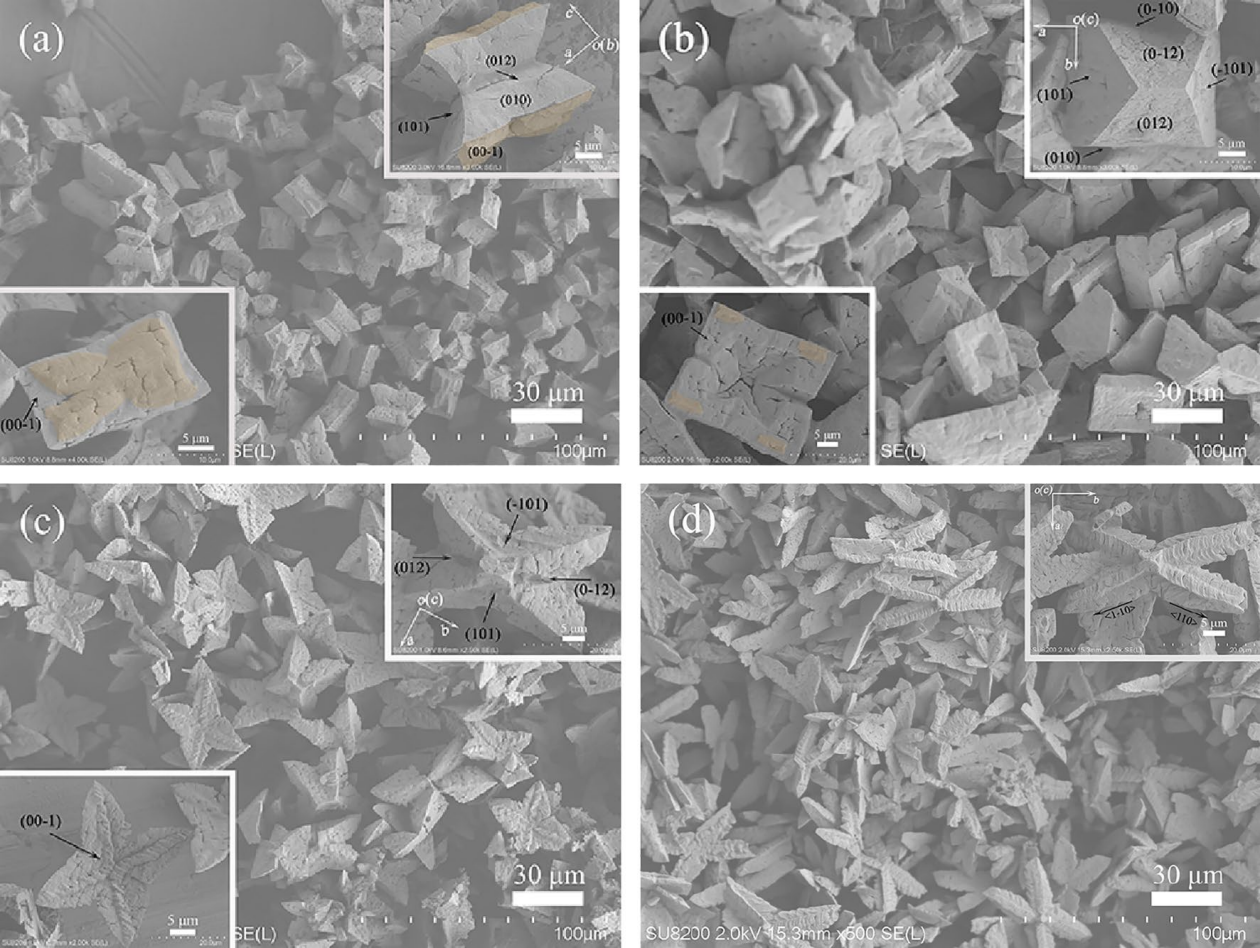
Representative FESEM images of the samples biomimetically synthesized for 60 (**a**), 90 (**b**), 130 (**c**), or 180 min (**d**) with the supernatant.

To further verify this viewpoint, the time-course biomimetic mineralization experiments with the supernatant were performed. Figure 6 presents the typical FESEM images of the products. At 60 min of mineralization, the products all exhibited a twin crystal structure composed of coffin-like individuals, and an overgrowth structure can be seen on the (00-1) face of each individual (Fig. 6a and its insets). At this stage, the overgrown crystals appeared to be squashed and no longer had the smooth faces (lower left inset of Fig. 6a). For 90 min, the obtained crystals mainly possessed coffin-like single crystal habit (Fig. 6b and its upper right inset). Figure 6b lower left inset displays that evident pits were formed on (00-1) face of coffin-like crystal, and that four small pieces of the overgrown crystal were symmetrically distributed on the corners of its (00-1) face, indicating that the overgrown crystal dissolution significantly occurs. Further extending the reaction time to 130 min, the quadrangular tabular morphology was developed, and no overgrown crystal was visible (Fig. 6c and its insets). Note that substrate crystal {012}, {101} and {00-1} faces gradually expanded and substrate crystal {010} faces as well as overgrown crystals gradually disappeared over time, which are consistent with that in bacterial mineralization. Simultaneously, the profile of pH and NH_3_-N in the biomimetic solution (Supplementary Fig. S4) is also similar to that in Fig. 5. It is interesting that prolonging biomimetic mineralization time to 180 min led to the appearance of X-shaped structure (Fig. 6d), which has been not obtained in bacterial mineralization system (Fig. 2). It possibly results from the continuous diffusion of NH_3_ into the mineralization solution, resulting in the rise in pH and NH_4_^+^ concentration (Supplementary Fig. S4). The characteristic X shape has been obtained when struvite crystallized in the presence of urease-producing bacteria^11,27,36,37^. These further supported that the rise in pH and NH_4_^+^ facilitate the X-shape growth. Based on these results, it follows that the increases in pH and NH_4_^+^ content lead to the morphological evolution of bio-struvite.

### Mechanisms for the morphogenesis and evolution of struvite

**Figure 7:**
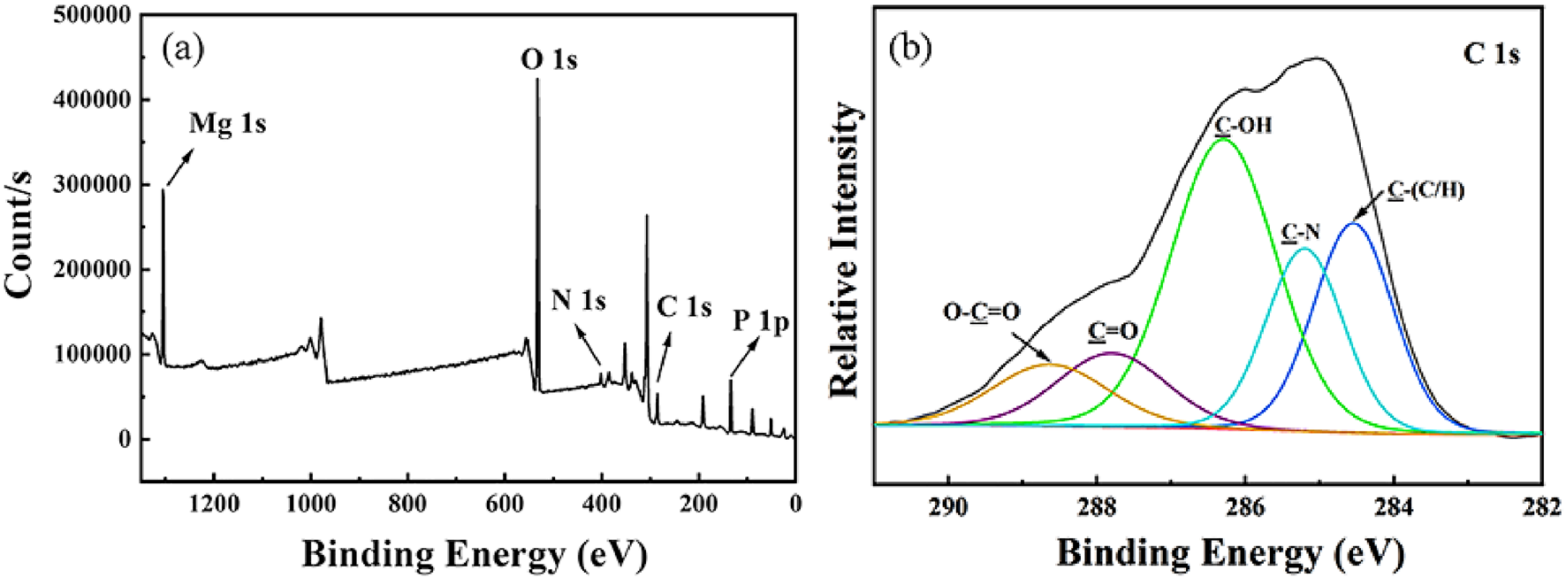
XPS wide survey scans (**a**) and high-resolution C 1 s (**b**) of the sample biomimetically synthesized for 30 min with the supernatant macromolecule component.

The bacterial mineralization experiments found that struvite with grouping structure (i.e., a small coffin-like crystal grown on a large trapezoid-like substrate crystal) can be obtained, and biomimetic experiments with different organic components confirmed that soluble macromolecule component secreted by strain H207 exert a key control role in the formation of the grouping structure. To further understand the mediation of the macromolecule component on struvite grouping morphogenesis, the FTIR and XPS techniques were used to characterize the struvite crystals obtained with the macromolecule component. The FTIR analyses showed that except for the characteristic vibration bands belonging to struvite, no organic functional groups were detected (e.g., Supplementary Fig. S5). This indicates that the organics content in the products was below the FTIR detection limit. However, the XPS results showed that the presence of carbon element in the samples (e.g., Fig. 7a), and the mass fraction of carbon up to 16.50%. Given that XPS is a surface-sensitive analysis technique, the organics is likely to be concentrated on the surfaces of struvite. Moreover, the zeta potential of the struvite was measured to be − 12.20, which significantly higher than that of pure struvite, i.e., − 19.42, further supporting that the organics was adsorbed on the surfaces of struvite (Supplementary Table S1). To identify the possible functional groups present in the organics, the C1*s* high-resolution spectrum was further deconvoluted, and the assignment and quantification of these functional groups were listed in Supplementary Table S2. As depicted in Fig. 7b and Supplementary Table S2, the C1*s* was resolved into four peaks, i.e., the peak at 284.3 eV (2.90%) can correspond to the C–(C/H) from lipids or amino acid side chains, the peaks at 285.0 (2.47%) to C–N from amine or amide, the peak at 286.1 eV (6.51%) to C–O–(H/C) from alcohol, ether, or phenol, and the peak at 287.8 (4.62%) to O–C=O from carboxylic acid, carboxylate, or ester^38–42^. As a result, the XPS analyses can assign the macromolecules anchored on the surfaces of struvite grouping as the compounds enriched in hydroxyl/ether, amine, and carboxyl groups. Among them, the electronegative carboxyl group has been reported to be able to strongly bind with metal ions including Mg^2+^ ^43,44^. In this respect, the formation of grouping structure can arise from the strong interaction between carboxyl group and the (00-1) face of the substrate crystal. As a hemimorphic crystal, the (001) face of struvite is terminated by NH_4_^+^ groups, but (00-1) face by PO_4_^3−^ and Mg(H_2_O)_6_^2+^ groups^45,46^. Therefore, the macromolecules with electronegative carboxyl group could be easy to bind Mg^2+^ ion on the (00-1) face, and the residual free carboxyl group in the macromolecules could also adsorb Mg^2+^ ions, acting as nucleation sites for the overgrown struvite. As a result, the small overgrown struvite crystals were induced to form on the larger substrate struvite crystals with trapezoidal shape (e.g., Fig. 2a).

**Figure 8:**
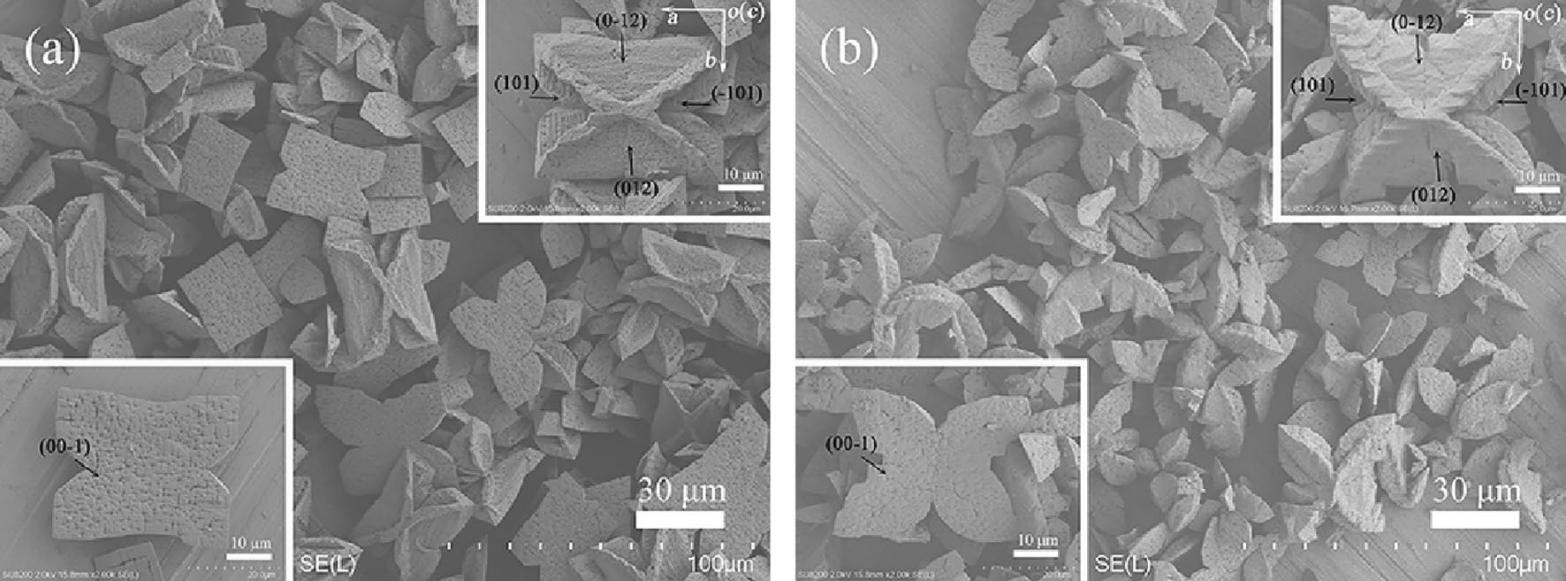
FESEM images of struvite biomimetically synthesized for 130 min in the presence of supernatant with adding 10 mmol/L phosphate and 3 mmol/L magnesium ions (**a**) or 7 mmol/L magnesium ions (**b**).

In terms of the morphological evolution of struvite, it is clear that two subunits of the primary crystal grouping underwent different evolution process. Specifically, the overgrown crystals gradually vanished with time, while the substrate crystals grew into the larger coffin-like, and quadrangular tabular morphologies (e.g., Fig. 2). The biomimetic experiments with the supernatant further supports this evolutionary process (e.g., Fig. 6a–c). It can be believed that the dissolution of overgrown crystals provides certain source of material for the growth and evolution of substrate crystals. For the evolution of trapezoid-like substrate crystals, it is clear that preferential growth along the *b* axis occurred. Wierzbicki et al*.*^47^ found that the growth of struvite along the [100] direction was significantly inhibited as the concentration of phosphocitrate increased, and their molecular modeling results showed that phosphocitrate had strong affinity to {101} faces. Here, the macromolecules in the initial medium could play a similar role, so that the crystals preferentially grew along the *b* axis, accompanied by the enhanced expressions of {101}, {00-1}, and {012} faces as well as weakened expressions of {010} faces. At the later growth period, however, the significant dissolution on the quadrangular tabular crystal faces arose (Fig. 2c,d). The biomimetic experiments found that the quadrangular tabular morphology can further evolve into X-shaped morphology by growing along four corners, i.e., the [110] and [1-10] directions (Fig. 6c,d), indicating that the substrate crystals underwent coupled dissolution–precipitation process at this stage and that the supersaturation is higher at the corners than the crystal surfaces. Meanwhile, one can find from Fig. 6b that the (00-1) face of substrate crystal dissolved prior to its {012} and {101} faces. In present system, the dissolution of substrate crystals can be attributed to the scarcity of phosphate at the later growth period due to the initial low phosphate content (Fig. 6 and Supplementary Table S1). Our molecular modeling results showed that the {00-1} face has a much higher density of PO_4_^3−^ than the {101} and {012} faces (Supplementary Fig. S6). It appears that the crystal face with higher phosphate content is preferentially dissolved, so as to provide the necessary phosphate ion for growth along the corners. To this end, the biomimetic experiments in the presence of the supernatant with high phosphate and low magnesium contents (i.e., adding 10 mmol/L phosphate and 3 or 7 mmol/L magnesium ions, respectively) were carry out. After 130 min of mineralization, the struvite both exhibited quadrangular tabular shape (Fig. 8a,b), and it can be clearly seen that the crystal {101} and {012} faces in each case visibly dissolved (Fig. 8a,b upper right insets), while bottom (00-1) face was smooth (Fig. 8a,b lower left insets). Therefore, it can be concluded that the initial low phosphate content leads to the preferential dissolution of (00-1) face with high phosphate content.

## Conclusion

Bacterial in situ mineralization experiments showed that the crystal grouping composed of large trapezoid-like substrate crystal and small coffin-like overgrown crystal can be obtained, and biomimetic experiments involving individual bacterial components revealed that macromolecules secreted by strain H207 exert a key control role in the formation of the grouping structure, i.e., the electronegative carboxyl group of macromolecules can strongly bind with Mg^2+^ ion on the (00-1) face of substrate crystal, and the residual free carboxyl group in the macromolecules can further adsorb Mg^2+^ ions, acting as nucleation sites for the overgrown struvite. With extending bacterial incubation time, the overgrown crystal gradually dissolved and disappeared, while the large substrate crystal further evolved from trapezoidal shape through coffin-like to quadrangular tabular structures, with firstly growing along the [010] direction and subsequently the [110] and [1-10] directions, and the time-course biomimetic experiments with supernatant testified that morphological evolution of strain H207-induced struvite resulted from the increase of pH and NH_4_^+^ content caused by the bacterial metabolism of nitrogenous organic matter. In a word, current work is useful for a better understanding of struvite biomineralization.

## Materials and methods

### Materials

All starting chemicals were obtained commercially without further purification. Magnesium chloride hexahydrate (MgCl_2_·6H_2_O), sodium hydroxide (NaOH), sodium dihydrogen phosphate (NH_4_H_2_PO_4_), triammonium phosphate ((NH_4_)_3_PO_4_)), ammonia water (NH_3_·H_2_O), hydrochloric acid (HCl), and glucose are of analytical grade and purchased from Sinopharm Chemical Reagent Co., Ltd. The yeast extract and malt extract are of biotech grade and purchased from Oxoid Ltd. and BBI Life Sciences Ltd., respectively. Deionized water was used in all of the experiments.

### Bacterial cultivation and metabolic component separation

A halophilic actinomycetes *Microbacterium marinum* sp. nov. H207, isolated from deep sea water, was used in present study^48^. The strain was maintained in modified ISP2 medium with the following components: 3 g/L glucose, 3 g/L malt extract, and 12 g/L yeast extract. The initial pH value of the medium was adjusted to 7.50 with 5 mol/L NaOH solution, and the medium was then autoclaved for 20 min at 121 °C. For metabolic components separation, strain H207 was cultured aerobically in 100 mL medium for 72 h at 30 °C with constant shaking (200 rpm). The as-obtained medium was then centrifuged at 10,000 rpm for 20 min to obtain the pellet bacterial cells, and the harvested cells were rinsed three times with sterilizing deionized water to remove residual growth medium. The resultant supernatants were filtered through 0.22 μm cellulose acetate membranes to eliminate any remaining cell debris. Subsequently, the ultrafiltration method was employed to further divide the supernatants into low molecular-weight (LMW) (< 5000 Da) and macromolecule components according to our previous study^16,24^. After ultrafiltration, the macromolecule component of the supernatant was washed twice with deionized water. A portion of unseparated liquid culture, filtered supernatant, supernatant LMW and macromolecule components, as well as harvested native cells were used in following biomimetic mineralization experiments.

### Bacterial mineralization

In a typical bacterial mineralization run, 2 mL of 1 mol/L MgCl_2_ solution was added into 98 mL of the modified ISP2 medium by filtration sterilization with 0.22 μm cellulose acetate membranes to reach a final Mg^2+^ concentration of 20 mmol/L. Subsequently, the medium was inoculated with 0.1 mL of seed culture, and cultured aerobically at 30 °C with constant shaking at 200 rpm. After given incubation time, the mineralized products were separated by natural sedimentation, washed three times with 100% absolute ethyl alcohol, and dried at room temperature for 24 h. All the experiments were performed in triplicate.

### Biomimetic mineralization

The biomimetic growth of struvite was conducted in an ammonia diffusion system at 30 °C. In a typical procedure, 1 mL of 1 mol/L MgCl_2_ solution was added into a beaker containing 49 mL of the solution with different bacterial components, which were separated from 49 mL of culture according to the procedures described in Sect. 2.2. The pH of the solution was adjusted to 7.5 using a 5 mol/L NaOH/HCl solution. After that, the beaker was covered with stretched parafilm into which six needle holes were punched, and placed into a closed desiccator. A bottle (30 mL) of diluted ammonia water (3 mol/L) was also placed in the desiccator as a source of ammonia. Especially, for the experiments with bacterial cells or supernatant macromolecule component, 0.12 mmol of (NH_4_)_3_PO_4_ was added to the solution (reaching a 2.4 and 7.2 mmol/L of phosphate and ammonium concentration, respectively) for matching the concentrations in the supernatant (Supplementary Table S3). The control experiments with uninoculated culture medium were also carried out. All the experiments were run in triplicate. To avoid microbial contamination, the experimental equipments and solutions were UV-sterilized for 30 min in a clean bench prior to the experiments, and the experiments were also conducted in the clean bench. After a given mineralization time, the precipitated products were separated by natural sedimentation, washed three times with 100% absolute ethyl alcohol, and dried at room temperature for 24 h.

### Analytical techniques

The pH of the medium was measured with a pH meter (Inolab WTW series pH 740). The NH_3_-N concentration was measured at 640 nm on the UV–Visible spectrophotometer (TU-1901, Beijing Purkinje General Instrument Co., Ltd.) according to the protocol given by Solórzano^49^. The total phosphate content was measured on the same spectrophotometer with 827 nm using the ascorbic acid method, as described by Murphy and Riley^50^. The structure and phase composition of the products were analyzed by X-ray powder diffraction (XRD) with a Japan Rigaku SmartLab X-ray diffractometer equipped with graphite-monochromatized Cu Kα radiation (λ = 0.154056 nm), at a scanning rate of 0.02° s^−1^ in the 2θ range of 10°–50°. The morphology and texture of the precipitates were studied using a HITACHI SU8220 field emission scanning electron microscope (FESEM), with the operation acceleration voltage of 2 or 3 kV. Infrared spectra were taken on the samples that were pelletized with KBr powder by using a Nicolet Impact 8700 Fourier transform infrared (FT-IR) spectrometer in the wavenumber range 4000–400 cm^−1^. X-ray photoelectron spectra (XPS) were obtained on a Thermo-ESCALAB 250 X-ray photoelectron spectrometer using AlKa radiation over the energy range of 0–1350 eV. The zeta potential of the samples was determined by NanoBrook Omni Zeta Potential Analyzer (Brookhaven Instruments Corporation, U.S.A.). Models of crystal surfaces of struvite were constructed using Materials Studio 2017.

## Supplementary information

Supplementary Informations.
