# Supplementary material for: Morphogenesis and evolution mechanisms of bacterially-induced struvite
Source: Sci Rep. 2021 Jan 8;11:170. doi: 10.1038/s41598-020-80718-y (PMC7794283; doi:10.1038/s41598-020-80718-y)
Supplement: Supplementary file 1 — Supplementary Informations. [file 41598_2020_80718_MOESM1_ESM.pdf]

Morphogenesis and evolution mechanisms of bacterially-induced struvite

Tian-Lei Zhao[1], Han Li[1], Hao-Fan Jiang[1], Qi-Zhi Yao[2*], Ying Huang[3], Gen-Tao Zhou[1,4*]

[1] CAS Key Laboratory of Crust-Mantle Materials and Environments, School of Earth and Space Sciences, University of Science and Technology of China, Hefei 230026, P. R. China.

[2] School of Chemistry and Materials Science, University of Science and Technology of China, Hefei 230026, P. R. China.

[3] State Key Laboratory of Microbial Resources, Institute of Microbiology, Chinese Academy of Sciences, Beijing 100101, China.

[4] CAS Center for Excellence in Comparative Planetology, Hefei 230026, University of Science and Technology of China, P. R. China.

Corresponding author: Prof. Dr. Gen-Tao Zhou

Tel.: 86 551 63600533

Fax: 86 551 63600533

Email: gtzhou@ustc.edu.cn

Supplementary Materials:

Supplementary figures and figure legends

Supplementary references

Supplementary tables

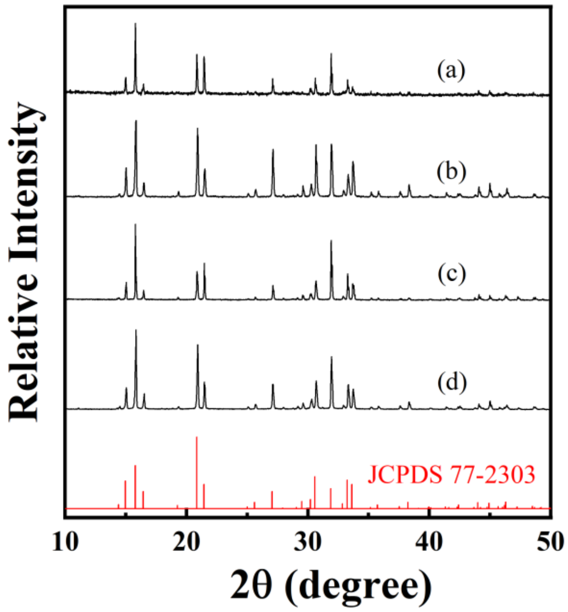

Figure S1 Representative XRD patterns of the samples biomimetically synthesized for 30 min with unseparated liquid culture (a), bacterial cells (b), supernatant (c), or uninoculated culture medium (d).

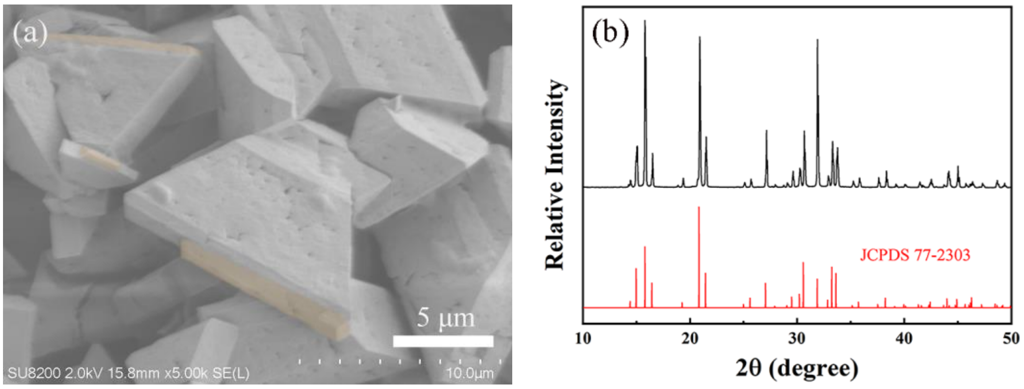

Figure S2 Representative FESEM image (a) and XRD pattern (b) of the sample biomimetically synthesized for 2 h with unseparated liquid culture in the presence of 1 mol/L ammonia water. The overgrown crystals were colored with orange.

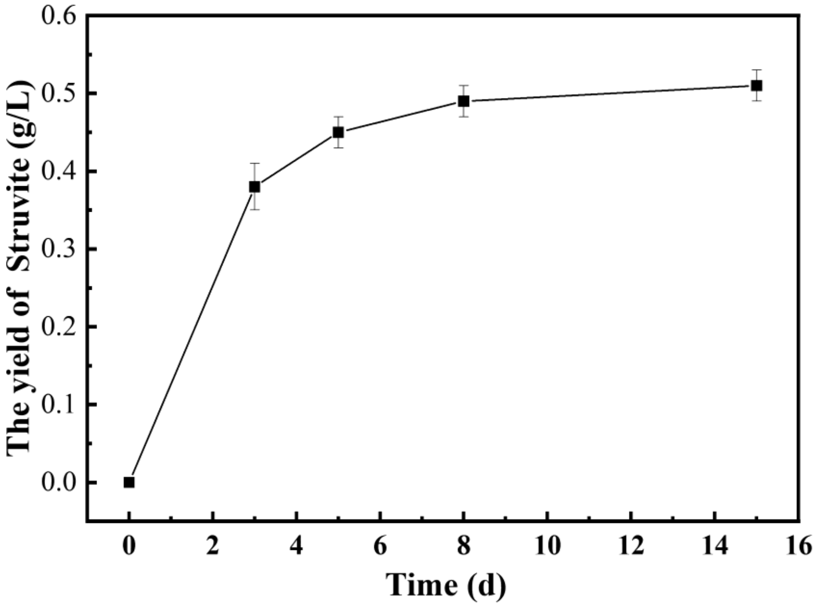

Figure S3 Time evolution of struvite yield obtained by bacterial mineralization

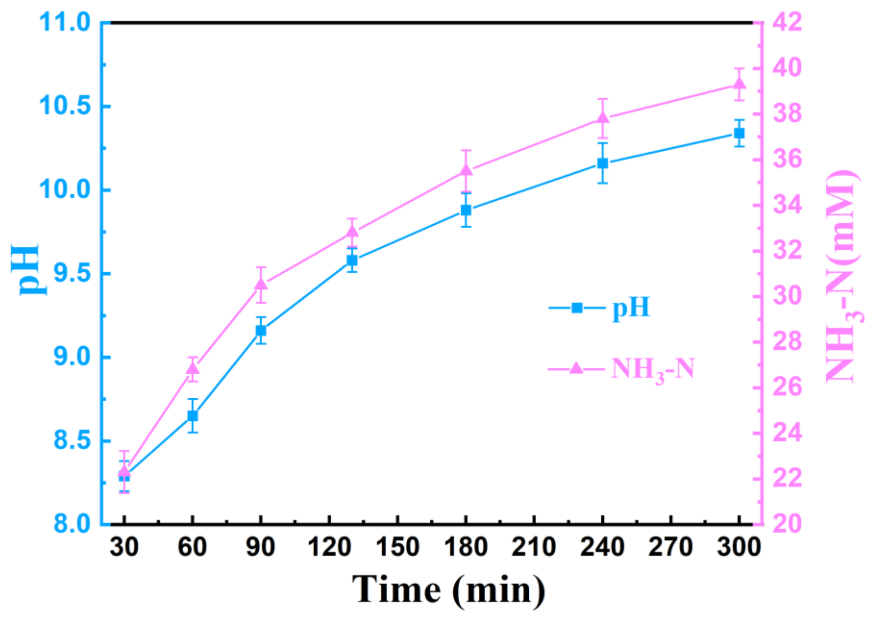

Figure S4 Time evolution profile of pH and NH_3-N content in biomimetic mineralization experiment with the supernatant.

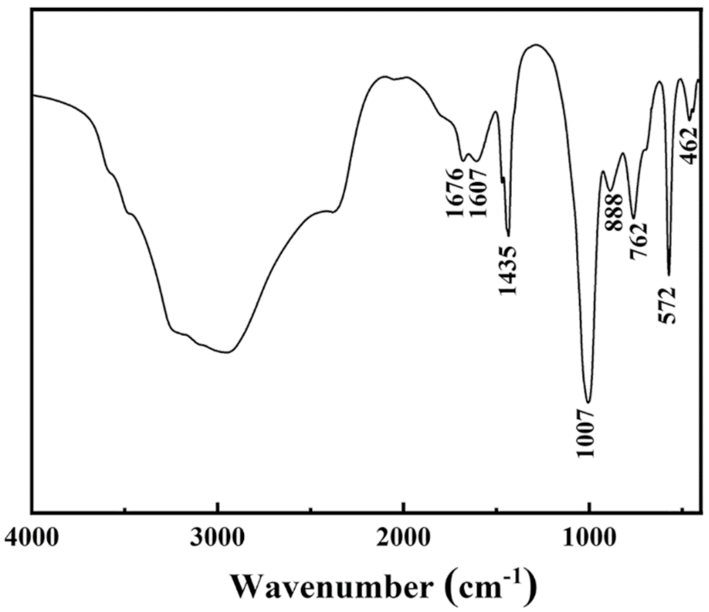

Figure S5 FT-IR spectrum the sample biomimetically synthesized for 30 min with the supernatant macromolecule component.

The absorption occurring between 3600 and 2800 cm^{-1} can be attributed to the stretching vibration of hydroxyl groups and the symmetric/antisymmetric stretching vibration of N-H in the NH_4^+ group, the bands appearing at 1676, 1607, and 1435 cm^{-1} are caused by the NH_4^+ antisymmetric bending, the peaks centered at 1007, 572, and 462 cm^{-1} can be ascribed to antisymmetric bending, asymmetric variable angle vibration, and symmetric variable angle vibration, respectively, and the peaks at 884/759 cm^{-1} correspond to ammonium-water/water-water H bonding. In all, the IR vibrational bands were good in agreement with pure struvite reported elsewhere[1,2], and no organic functional group was detected.

References

1. Kurtulus, G. & Tas, A. C. Transformations of neat and heated struvite ($MgNH_4PO_4 \cdot 6H_2O$). *Mater. Lett.* 65, 2883-2886 (2011).
2. Moulessehoul, A., Gallart-Mateu, D. & Harrache, D. Conductimetric study of struvite crystallization in water as a function of pH. *J. Cryst. Growth* 471, 42-52 (2017).

Table S1 The zeta potential of the struvite samples measured at pH 9.

Struvite obtained without macromolecules	Struvite obtained with macromolecules
-19.42 ± 0.74	-12.20 ± 0.58

Table S2 Binding energies (eV) and quantization of XPS C 1s spectral bands.

Mass fraction of C (%)	16.50			
Peak (eV)	284.3	285.0	286.1	287.8
Functional group	C-H/C	C-N	C-OH	O-C=O
Percentage (%)	2.90	2.47	6.51	4.62

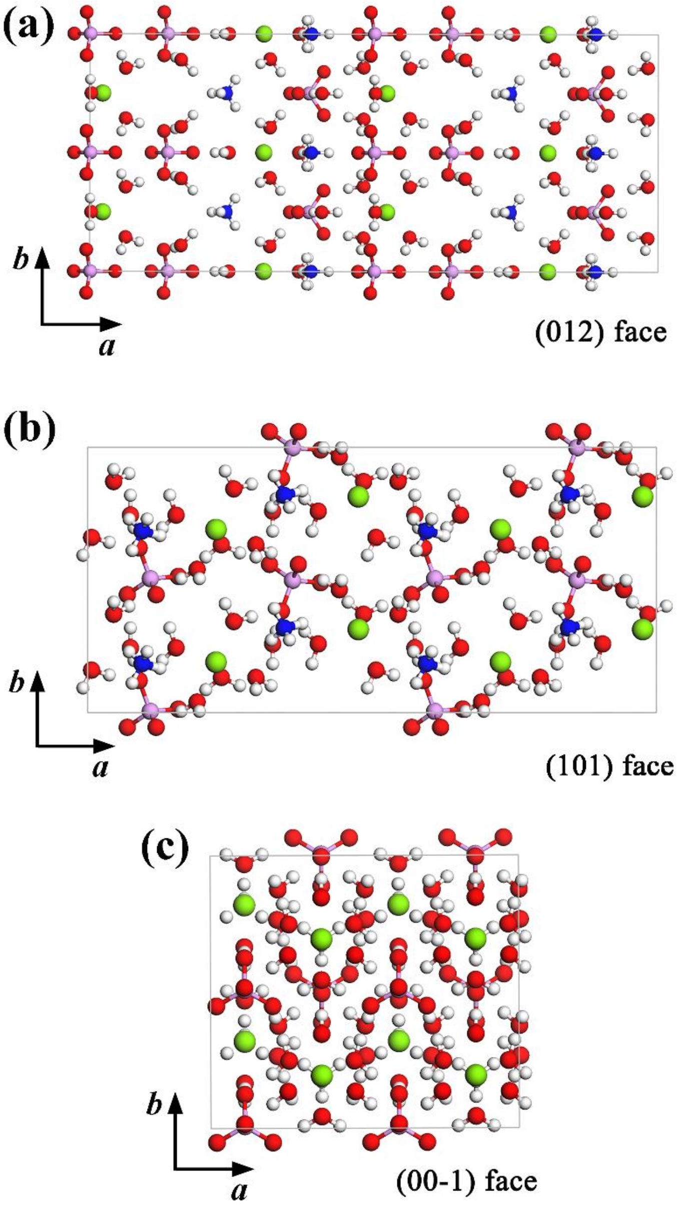

Figure S6 Crystal surfaces of struvite, including (012) with the 2×2 supercell parameters of a = 33.271 Å, b = 13.910 Å (a) (101) 2×2 supercell, a = 26.398 Å, b = 12.284 Å (b), (00-1) 2×2 supercell, a = 13.910 Å, b = 12.284 Å (c) with PO_4^{3-} content of 2.871, 3.074, and 5.856 μmol/m^2, respectively. The atoms are denoted by the balls of the following colors: Mg, green; P, purple; N, blue; O, red; H, white.

The struvite unit cell obtained by Whitaker and Jeffery[3] was adopted here. Firstly, 2×2×2 supercell with parameters of a = 13.882 Å, b = 12.274 Å, and c = 22.398 Å was constructed. Subsequently, the crystal face slabs were constructed. Note that the atoms in the crystal are

periodically arranged in a certain direction, a repeating unit was chosen for (101) or (012) face construction. For the (00-1) face, it has been reported that the (001) face of struvite is terminated by NH_4^+ groups while the (001) face is terminated by PO_4^{3-} and $Mg(H_2O)_6^{2+}$ groups[4], thus a repeating unit with removing surface NH_4^+ was chosen here.

The number PO_4^{3-} on each crystal face within the 2×2 supercells was counted from Figure S5, respectively. The PO_4^{3-} content on each crystal face is calculated according to the following equation:

$$PO_4^{3-} \text{ content} = N_{PO4}/(NA \times a \times b)$$

where N_{PO4} is the number of or PO_4^{3-} on each face within the 2×2 supercells, a and b are corresponding cell parameters of the 2×2 supercell, NA is Avogadro's constant.

References

3. Whitaker, A. & Jeffery, J. W. The crystal structure of struvite, $MgNH_4PO_4$. $6H_2O$. *Acta Crystallogr., Sect. B: Struct. Crystallogr. Cryst. Chem.* 26, 1429-1440 (1970).
4. Abbona, F., Calleri, M. & Ivaldi, G. Synthetic struvite, $MgNH_4PO_4{\cdot}6H_2O$: correct polarity and surface features of some complementary forms. *Acta Crystallogr., Sect. B: Struct. Sci.* 40, 223-227 (1984).

Table S3 The concentrations of NH_3-N and phosphate of uninoculated culture medium and the inoculated medium for 3 day without Mg^{2+}.

Incubation time (d)	NH_3-N concentration (mM)	PO_4^{3-}-P concentration (mM)
0	4.03	3.53
3	7.28	2.47
